# Associative correlation between clinical manifestations of PICIS and CRID: modern trends in the pre-early diagnostics

**DOI:** 10.1186/1878-5085-5-S1-A130

**Published:** 2014-02-11

**Authors:** Kusum A Akhmedilova, Murat M Agirov, Zhamilya A Tabaksoeva, Natalia E Cherepakhina, Tatyana A Bodrova, Zaur S Shogenov, Sergey V Suchkov

**Affiliations:** 1 I.M. Sechenov First Moscow State Medical University (FMSMU), Moscow, Russia; 2 Russian State Medical University (RSMU), Moscow, Russia; 3 Federal Agency of Medical and Biological Problems under the Government of the Russian Federation, Moscow, Russia; 4 Department of Economy of Health Services, The Higher School of Economy, Moscow, Rusia

## 

The association between microbial landscapes (*microbiome*) and various immunopathological states with postinfectious clinical-and-immunological syndrome (PICIS) can be correlative or causal. In patients with chronically relapsing infectious diseases (CRID), syndromal forms of the immune-mediated disorders depend crucially on the stage of the inflammatory process occurring in the targeted organs or tissues and the overall chronization of the disease.

For example, early stages of CRID are concomitant with postinfectious secondary immunodeficiency syndrome (PIFSI) (› 50%), whereas the incidence and thus the contribution of postinfectious autoimmune syndrome (PIFA) and autoimmune syndrome associated with postinfectious secondary immunodeficiency (PIFASID) do not exceed 20%. At the subsequent stages, the clinical manifestations are different, viz., the incidence and thus the contribution of the autoimmunity rises dramatically (to 50% at the intermediate stages (PIFA) and to 60% at the final stage (PIFASID).

The correlation between the stage of CRID and the form of PICIS is also characterized by the involvement of an additional (the third) component, viz., a clinical form of CRID. Here are several analytical examples related to:

**(1) clinical form of CRID:** in patients with primary pyelonephritis (PPNP) and infectious myocarditis (IM), PIFSI is detected in 75% of clinical cases, whereas in patients with secondary pyelonephrites (SPNP) and autoimmune myocardites (AIM) the contribution of PIFSI is notably reduced (to 25%) giving way to the autoaggression (the incidence and thus the contribution of PIFA and PIFASID increases to 60% and 85%, respectively);

**(2) stage of CRID:** at the early (including preclinical ones) stages (< 3 months for CPN and < 1 month for myocarditis (M)), PIFSI is detected in 40% of cases; however, at the advanced stages of CRID its incidence reduced appreciably, while that of autoimmune syndromes increases in contrast;

**(3) rate of progression and chronization of CRID:** in patients with relapsing or rapidly progressing CRID (e.g., ICIIP or AIM), the incidence and thus the contribution of PIFSI do not exceed 32-36%, while the share of autoimmune syndromes reaches 80-100%. In such patients, persistent forms of chronic meningoencephalitis (e.g., ICIIP) or AIM associated with myocardial dystrophies are predominant.

These findings suggest that PIFSI is not only the outcome of the infectious process, but also the predictive sign to promote chronization of the disease and to represent a factor responsible for getting the clinical course chronic. Further progression and *chronization* of CRID are controlled by postinfectious autoaggression factors, such as PIFA and PIFASID which are thus also considered as predictive factors to monitor transformation of subclinical forms into the clinical ones.

